# NSCs Under Strain—Unraveling the Mechanoprotective Role of Differentiating Astrocytes in a Cyclically Stretched Coculture With Differentiating Neurons

**DOI:** 10.3389/fncel.2021.706585

**Published:** 2021-09-24

**Authors:** Jella-Andrea Abraham, Stefan Blaschke, Samar Tarazi, Georg Dreissen, Sabine U. Vay, Michael Schroeter, Gereon R. Fink, Rudolf Merkel, Maria A. Rueger, Bernd Hoffmann

**Affiliations:** ^1^Mechanobiology, Institute of Biological Information Processing (IBI-2), Research Centre Juelich, Juelich, Germany; ^2^Department of Neurology, Faculty of Medicine and University Hospital, University of Cologne, Cologne, Germany; ^3^Cognitive Neuroscience, Institute of Neuroscience and Medicine (INM-3), Research Centre Juelich, Juelich, Germany

**Keywords:** neural stem cell, astrocyte, NSC differentiation, mechanoresponse, cyclic strain, cortical neuron, mechanoprotection, neural stem cell niche

## Abstract

The neural stem cell (NSC) niche is a highly vascularized microenvironment that supplies stem cells with relevant biological and chemical cues. However, the NSCs’ proximity to the vasculature also means that the NSCs are subjected to permanent tissue deformation effected by the vessels’ heartbeat-induced pulsatile movements. Cultivating NSCs under common culture conditions neglects the—yet unknown—influence of this cyclic mechanical strain on neural stem cells. Under the hypothesis that pulsatile strain should affect essential NSC functions, a cyclic uniaxial strain was applied under biomimetic conditions using an in-house developed stretching system based on cross-linked polydimethylsiloxane (PDMS) elastomer. While lineage commitment remained unaffected by cyclic deformation, strain affected NSC quiescence and cytoskeletal organization. Unexpectedly, cyclically stretched stem cells aligned in stretch direction, a phenomenon unknown for other types of cells in the mammalian organism. The same effect was observed for young astrocytes differentiating from NSCs. In contrast, young neurons differentiating from NSCs did not show mechanoresponsiveness. The exceptional orientation of NSCs and young astrocytes in the stretch direction was blocked upon RhoA activation and went along with a lack of stress fibers. Compared to postnatal astrocytes and mature neurons, NSCs and their young progeny displayed characteristic and distinct mechanoresponsiveness. Data suggest a protective role of young astrocytes in mixed cultures of differentiating neurons and astrocytes by mitigating the mechanical stress of pulsatile strain on developing neurons.

## Introduction

Neural stem cells (NSC) are the source of all neurons and glial cells in the central nervous system. NSCs sense chemical and mechanical cues located in their specialized extracellular environment, referred to as the neural stem cell niche. In this specialized microenvironment, NSCs and progenitor cells are supplied by numerous extracellular signals that regulate stem cell characteristics (for review see Miller and Gauthier-Fisher, 2009). As an essential feature of the niche, its close proximity to vasculature modulates and controls characteristic NSC functions, i.e., proliferation and differentiation (Goldberg and Hirschi, 2009; Otsuki and Brand, 2017; Karakatsani et al., 2019).

Due to blood pulsation, brain tissue is continuously in motion with tissue deformation approaching amplitudes of up to 30% (Drew et al., 2011). Thus, while often overlooked, ubiquitous mechanical cues affect the niche, such as the stiffness of the surrounding microenvironment (Blaschke et al., 2019) or topographical cues (Baek et al., 2018) that arise due to the complex architecture of the microenvironment and its interconnected network of extracellular proteins. Furthermore, substrate stiffness interferes with cell cycle regulation inducing cell quiescence (Blaschke et al., 2019). Strain as a mechanical cue to influence NSCs was reported by Arulmoli and colleagues (Arulmoli et al., 2015). With a device that supplied equibiaxial strain, the authors exposed mice NSCs to 10% of static strain and observed a stretch-induced reduction in oligodendrocyte regeneration. Another study reported a change of the substrate’s physical property, i.e., conductivity, after stretch as a cue to tune stem cell lineage commitment to promote neuronal differentiation (Srivastava et al., 2013).

Furthermore, mechanical strain exerted by pulsating vessels may substantially impact NSCs. We and others previously showed that mechanical factors essentially affect the mode of differentiation and lineage commitment (Engler et al., 2006; Yokota et al., 2009; Baek et al., 2018; Blaschke et al., 2019). However, little is known about the role of cyclic strain as a cue for NSCs fate. We previously showed that such pulsating movements could be mimicked *in vitro* by cultivating cells on top of a stretchable elastomer (Faust et al., 2011; Noethel et al., 2018; Abraham et al., 2019). Using this system, cyclically stretched neurons exhibited neuronal outgrowth in a perpendicular direction to the uniaxial strain (Abraham et al., 2019). Such cellular reorientation is a characteristic response of most mammalian cells upon a stretch. This way, actin cytoskeleton’s reorientation perpendicular to the strain direction is consecutively followed by the other cytoskeletal systems and by cell shape reorientation (Faust et al., 2011; Zielinski et al., 2018; Springer et al., 2019). Notably, cyclic stretch induced outgrowth of neuronal branches, suggesting a positive mechanical strain involvement in developmental processes (Abraham et al., 2019).

We here explore the effect of cyclic uniaxial strain on primary rat NSCs and their young progeny.

## Material and Methods

### Primary Neural Stem Cell Culture

For NSC cultivation, cell culture dishes were pre-coated with a 15% L-poly-ornithine (Sigma Aldrich, St. Louis, USA) solution overnight. Due to the toxicity of soluble poly-L-ornithine, plates were washed three times with PBS. Bovine fibronectin (R&D Systems, Minneapolis, MN) solution in PBS (2.5 mmol/L) was added to the plates and incubated for 2 h. NSCs were isolated from pregnant Wistar rats at 13.5 days of gestation (animal testing license: 81-02.04.2018.A90, LANUV NRW, Germany) following previously published protocols (Rueger et al., 2010; Blaschke et al., 2019). In brief, after decapitation, the embryo chain was removed and isolated from its placenta. The meningeal layer was removed, and hippocampal tissue was detached. The cortices were pipetted up and down in fresh media to mechanically dissect the cells from the tissue. After dissipation, the remaining tissue clumps of the two hemispheres were allowed to settle for 1 min. Only the upper supernatant, containing dissipated NSCs, was transferred to a pre-coated cell culture dish in DMEM/F12 medium (Life Technologies, Darmstadt, Germany) plus 1% N2 supplement (Gibco, Karlsruhe, Germany), 1% penicillin/streptomycin, 0.6 mM L-glutamine, and 1% sodium pyruvate. Fibroblast growth factor (FGF, 10 ng/ml, Invitrogen, Karlsruhe, Germany) was added to ensure that cells remain their stemness throughout experiments unless stated otherwise. NSC culture dishes were supplied with the mitogen FGF every day. Media was changed every second day. A continuous FGF supply was pivotal to repress differentiation and maintain a homogenous population of rapidly dividing cells. For stretch experiments, NSCs were used from the second till the fifth passage. When cultivated on elastomer chambers, a density of 23,000 cells/cm^2^ was plated for experiments with NSCs. A number of 40,000 cells/cm^2^ were plated for experiments during cell differentiation.

### Primary Cortical Neuron Isolation

CO_2_-anesthetized pregnant rats (Wistar, Charles River, Sulzfeld) at 18–19 days of gestation were decapitated. Primary cortical cell isolation was performed as previously published (Abraham et al., 2019). In brief, the uterus with the embryo chain was removed from the mother rat, the placenta opened, and embryos were sacrificed *via* cervical dislocation. After cortical isolation, the tissue was placed in an ice-cold 0.05% trypsin-EDTA solution (Thermo Fisher Scientific, Waltham, USA) followed by incubation for 15 min at 37 °C. The trypsinized tissue was further transferred to a pre-warmed neurobasal cell culture medium (Thermo Fisher Scientific, Waltham, USA) supplemented with B27 (Thermo Fisher Scientific, Waltham, USA), Gentamicin (Sigma, Taufkirchen, Germany), and GlutaMAX (Thermo Fisher Scientific, Waltham, USA). Cortices were washed to remove residual trypsin and dispersed by pipetting up and down. The chambers were precoated over night with an avidin solution in water (1 mg/ml, Thermo Fisher Scientific, Waltham, USA). For cocultures with astrocytes 20,000 cells/cm^2^ were plated on PDMS stretching chambers whereas 30,000 cells/cm^2^ were used for pure cultures. Cells were allowed to attach to the elastomer substrates at least for 4 h before the stretch experiments.

### Postnatal Astrocyte Cell Culture

Astrocytes were isolated from cortices of neonatal Wistar rats (P1 to P3). After decapitation cortical tissue was removed, hackled by a blade and then trypsinized at 37°C and 5% CO_2_ for 15 min (1% trypsin, 0.02% EDTA). After trypsinization, cells were transferred to pre-warmed astrocyte culture media containing DMEM (Thermo Fisher Scientific, Waltham, USA), supplemented with L-glutamine (2 mM, PAN-Biotech, Aidenbach, Germany), 1% penicillin-streptavidin (PAN-Biotech, Aidenbach, Germany), and 10% fetal bovine serum. After washing the tissue with culture media, the tissue was dissociated by pipetting up and down and centrifuged at 250 *g* for 2 min. Medium was exchanged 2 days after isolation. The cell culture contained astrocytes but also microglia. To get a cell culture of pure astrocytes, 7–10 days after isolation, microglia were gently removed by shaking the cell culture flask for 3 h at 250 rpm. Astrocytes were trypsinized for 10 min and subsequently centrifuged for 5 min at 250 *g*. To guarantee cell attachment, elastomer chambers were pre-coated with 1 mg/ml avidin and rested for 3 days before stretch experiments or adding primary cortical cells for coculture experiments. For stretch experiments with only postnatal astrocytes, a number of 30,000 cells/cm^2^ was plated on the chambers. Cocultures contained different numbers of astrocytes, while the number of neurons was kept constant with 20,000 cells/cm^2^.

### Elastomer Chamber Fabrication

A two-component polydimethylsiloxane (PDMS) based formulation (Sylgard 184, Dow Corning, Wiesbaden, Germany), was used to fabricate soft elastomer chambers as described elsewhere in detail (Faust et al., 2011; Abraham et al., 2019). Cross-linker and base polymer were mixed at a ratio of 1:40 (by weight) for 10 min and then degassed in a desiccator to remove air bubbles. The mixture was cast in molds and cured at 60°C for 16 h, resulting in elastomer substrates with a stiffness of 50 kPa. These chambers were square-shaped and exhibited a cell culture area of 4 cm^2^ and a media volume capacity of 550 μl. Elasticity measurements were performed as described before (Ulbricht et al., 2013). Before chamber coating (see above) and cell seeding, chambers were washed with isopropanol and mounted in chamber holders. The chamber holder with the mounted elastomer chamber was kept at 37°C for at least 6 h under sterile conditions to ensure full evaporation of isopropanol.

Chambers with a stiffer substrate used for the rotation experiments were fabricated by using a different PDMS based system (Sorta clear, Smooth-On, Macungie, USA). Here chambers were fabricated as described above with an exception of the curation step which was performed in room temperature for 16 h. The Sorta clear chambers had a stiffness of 330 kPa and were coated with a 50 kPa Sylgard 184 PDMS layer to achieve comparable culture conditions.

### Cyclic Stretch Experiments

For stretch experiments, elastomeric chambers were mounted with chamber holders in an in-house developed stretcher apparatus. The stretcher apparatus had a motorized stage and is controlled by an in-house developed software that allows setting various strain parameters. The chambers were stretched uniaxially with an amplitude of 15% and a frequency of 300 mHz (trapezoidal approximation of sine wave). This stretch protocol has been shown to induce a clear mechanoresponse of cortical neurons (Abraham et al., 2019). All stretch experiments were performed under sterile conditions. After stretching, cells were fixed with 4% paraformaldehyde (Electron Microscopy Sciences; Hatfield, PA, United States) in PBS. As a control, cells were cultivated in elastomer chambers without mechanical deformation. NSCs were allowed to adhere to the elastomer substrates for at least 4 h before stretch experiments started for indicated times. For lineage commitment experiments, cells were allowed to adhere for 24 h with FGF supplemented media. Afterwards, media were changed to FGF free media to induce differentiation. Cyclic stretch was initiated immediately after the media change for 5 days of continuously applied stretch. Media change was performed every day.

### Switching Strain Direction With Differentiating Astrocytes

Differentiating astrocytes were cultivated for 5 days with and without stretch. After 3 days of cyclic stretch, half of the stretched chambers were removed from the chamber holders, turned by 90 degrees, and mounted back to the holder. Subsequently, media was changed, and cells were stretched for additional 2 days before fixation. To avoid any mechanical damage during the handling of the elastomer chambers we used a different PDMS based system (Sorta clear, Smooth-On, Macungie, USA) that results in stiffer elastomer substrates. This system is less sensitive to small movements, but can still be stretched with the same stretch parameter as mentioned above. Sorta clear chambers had a stiffness of 330 kPa. The elastomer chambers were coated with a 50 kPa Sylgard 184 PDMS layer to achieve comparable culture conditions with the previously used elastomers.

### Immunocytochemistry

After cell fixation, cells were stained with Hoechst 33342 (1:500 Sigma-Aldrich, Louis, US) and primary antibodies against vimentin (1:500 mouse mAB ab92547 Abcam, Cambridge, UK), nestin (1:500 mouse MAB353, Sigma Aldrich, Louis, US), tubulin (1:500 rat MAB1864, Millipore, Germany), actin (1:500 Phalloidin Atto 488), Tuj-1 (mouse MAB1864, Millipore, Germany), GFAP (rabbit G9269, Sigma Aldrich, Louis, US), and Sox2 (1:200 goat AD2018, R&D Systems).

Before staining for BrdU, cells were incubated in 2 N HCl for 30 min for antigen retrieval. For visualization, fluorescein-labeled anti-mouse immunoglobin (goat anti-mouse IgG, Alexa Fluor TM 488, Thermo Fisher Scientific, Waltham, USA), anti-rabbit IgG (goat anti-rabbit IgG, Alexa Fluor TM 568, Thermo Fisher Scientific, Waltham, USA), anti-goat (donkey anti-goat IgG, Alexa Fluor TM 568, Thermo Fisher Scientific, Waltham, USA) were used. Cells were counted randomized and manually using ten pictures per trial taken with a Keyence BZ-9000 inverted fluorescence microscope (Keyence Osaka, Japan). Images were taken with a confocal laser scanning microscope (LSM880, Carl Zeiss, Germany) to investigate stress fibers. Imaging by confocal microscopy was also performed to image samples in three dimensions (Z-stack). For 3D reconstruction, Imaris software (Bitplane, Belfast, UK) was used.

### Cell Viability Assay

To assess whether mechanical strain influences the vitality of NSCs, dead cells were stained with propidium iodide (Life Technologies, Darmstadt, Germany) and counterstained, irrespective of viability, with Hoechst 33342 (Sigma Aldrich; St. Louis, USA) after 24 h of cyclic stretch or cultivated as a control. Images were taken with an upright microscope (Axiovert M2 imager, Carl Zeiss, Germany). Cells were counted randomized.

### RhoA Activation

NSCs were stretched for 24 h. Precisely 40 min before fixations, half of the stretched and control chambers were incubated with 30 μM lysophosphatidic acid (LPA, Santa Cruz, Texas, US). For cortical neurons and mixed cultures of neurons with astrocytes, cells were first grown as described above. Cells were then stretched for 24 h in the presence of LPA for the whole time of the last 40 min of stretching. Subsequently, cells were fixed as described above and labeled with Phalloidin Atto 488 and anti-tubulin antibody as described above.

### Bromodeoxyuridine Assay

NSCs were cultivated on elastomers for 24 h and exposed continuously to cyclic strain or cultivated on elastomers as a control. To assess the ratio of proliferating cells, 10 μM bromodeoxyuridine (BrdU; Sigma Aldrich; St. Louis, USA) was added to the cells 6 h before fixation to assess the ratio of proliferating cells. After immunocytochemical staining, fluorescence microscopy was performed as described above. A ratio of BrdU positive proliferating cells to all cells stained by Hoechst was assessed to compare proliferating cells across conditions. Ten random images per sample were taken, randomized, and counted manually.

### Live-Cell Imaging

Live-cell microscopy was performed with an upright microscope (Axiovert M2 imager, Zeiss, Germany). Cells were imaged using a dip-in objective (N-Achroplan 20× (NA 0.5), Zeiss, Germany) from above the chamber to avoid imaging through the relatively thick chamber bottom. Cells were observed while the elastomer chamber was stretched in steps of an amplitude of 4% and 0.5 mm/s velocity. After reaching 28%, the stretch was released from the system, and cells were again observed in the prestretch position. For analysis, cell branches were marked with the ImageJ software, and their lengths were calculated. For cell body analysis, the cell soma was marked with the ImageJ freehand drawing tool.

### Data Analysis

Images were analyzed using an in-house developed program (implemented in Python 3.7). Cytoskeletal filament orientation was determined at every single pixel. First, to analyze the orientation in the red and green channels, a binary mask for these channels was calculated. Thus, the image (red and green separately) was binarized using a local mean filter of 55 × 55 pixels (pixel size 0.27 μm) as a threshold. All gray values above this local threshold were defined as a signal, all others as background. A morphological opening was performed on this binary image using a disk structuring element with a radius of 2 pixels. Additionally, the same procedure was carried out using a local median filter (again 55 × 55 pixels). These two binary images were then combined into one mask. All nuclei were detected and removed from the mask to analyze structures outside of the cell nucleus only. For nuclei detection, the blue channel was used. Therefore, the mean gray value of the blue channel was calculated and multiplied by 2. This value was used as a threshold to separate the nuclei from the background. In the next step, the orientation was calculated for the red and green channels separately. First, the image was smoothed using a gaussian filter (filter size: sigma = 3 pixels). Each pixel’s orientation was then calculated using the structure tensor approach (Faust et al., 2011). Afterward, the orientation was further analyzed at the previously defined mask positions. Plots were obtained by considering the frequencies of pixel orientations, while the mean orientation was indicated as dashed lines. Only images taken from one experiment (with the same staining solutions) were used for the semi-quantitative analysis of cytoskeletal staining intensity. For this purpose gray values in arbitrary units were also extracted at all positions of the defined mask and averaged.

### Statistical Analyses

To analyze if the data were normally distributed, a one-sided Kolmogorov-Smirnov (KS) test was used. For not normally distributed data sets, a two-sided Mann-Whitney U test was performed. A one-sample t-test was used to test data sets against a hypothetical value of 100% when data were normalized for each experiment to the control. P-values are indicated with * for *p*-values < 0.05, ** for *p*-values < 0.01, and *** for *p*-values < 0.001. The number of independent experiments is indicated in the figure legends.

## Results

### Neural Stem Cells Are Deformed but Vital Under Cyclic Strain

**Figure 1:**
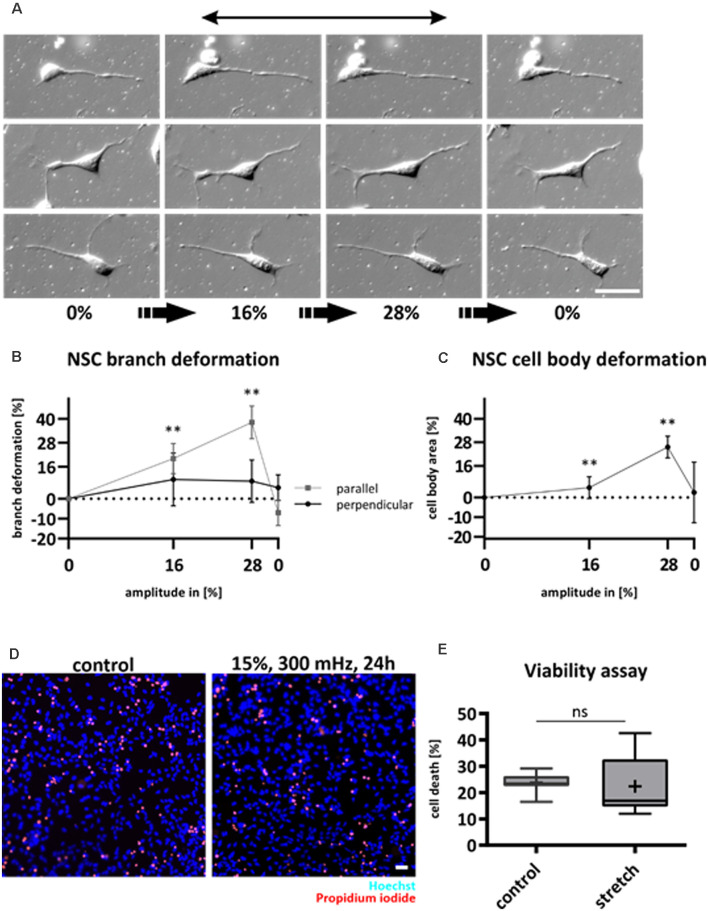
NSCs follow substrate deformation and are unaffected by cyclic strain. NSCs were stretched statically and observed *via* DIC live-cell microscopy to assess cell morphology changes caused by substrate **(A)**. Mechanical strain was increased stepwise by an amplitude of 4% and at a velocity of 0.5 mm/s. After reaching 28%, the stretch was released from the system. The black arrow indicates the strain direction. Deformation of NSC branches **(B)**. Deformation of NSC cell bodies **(C)**. The plots in **(B)** and **(C)** represent *n* = 5 chambers per parameter with at least 40 analyzed cells for each experiment. NSC branches that point in stretch or perpendicular direction were analyzed separately (p-value of parallel branches 0% vs. 16%: 0.008; 0% vs. 28% 0.008; p-value of cell bodies: 0% vs. 16%: 0.008; 0% vs. 28%: 0.008). Live/Dead assay of NSCs subjected to substrate strain compared to control **(D)**. Dead cells were stained with propidium iodide (red) and counted relative to the number of all cells stained with Hoechst (blue). The values are represented as box plots **(E)**, the black line shows the median, and the cross indicates the mean (*n* = 7 chambers from four different passages, ns = non-significant). Scale bars = 20 μm. *define significant differences (see “Material and Methods” section).

During migration, cytoskeletal structures have to continuously assemble, stabilize, and disassemble (Fletcher and Mullins, 2010). Due to the high cellular dynamics of migrating cells such as NSCs, we questioned the extent to which cyclic substrate strain will deform NSCs that are growing on top of the elastomers. To examine the immediate response to cyclic strain, NSCs were subjected to mechanical deformation while being observed *via* live-cell microscopy. The amplitude was increased stepwise by 4% until 28% of strain and imaged again in the release position (Figure 1A). Both cell soma and NSCs’ protrusions were deformed by substrate strain. Here, cell processes that aligned in the stretch direction followed substrate stretch (*p*-value < 0.01, Figure 1B), while a significant elongation of cell processes perpendicular to the stretch did not occur. On the same note, NSCs revealed stretched cell bodies by 25.5% after an applied substrate strain of 28% (Figure 1C).

NSCs were not negatively affected by substrate deformations as they did not retract and remained viable as assessed by propidium iodide (Figures 1D,E).

### Cyclically Stretched NSCs Remain More Quiescent without Altering Lineage Specification

**Figure 2:**
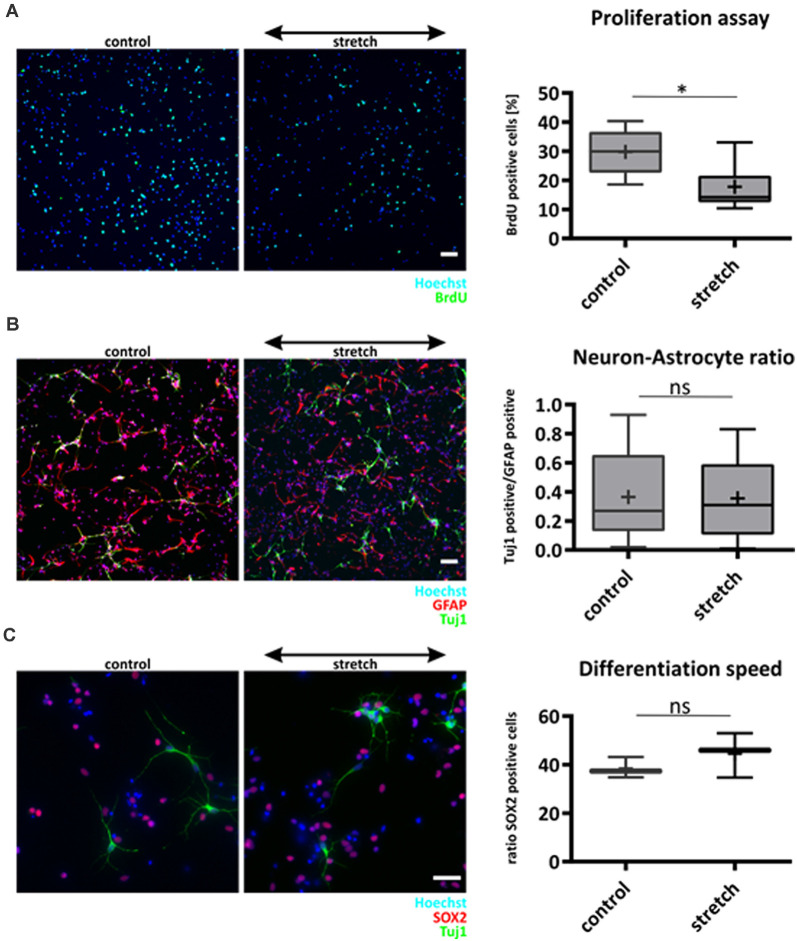
Effect of cyclic substrate deformation to stem cell characteristics of NSCs. Proliferation analysis after 24 h of cyclic strain **(A)**. The values are represented as box plots, the black line shows the median, and the cross indicates the mean (*p*-value = 0.021, *n* = 7 chambers per parameter from four different passages). Lineage commitment of NSCs when subjected to cyclic substrate deformation for 5 days during mitogen withdrawal **(B)**. Neuronal cells labeled against Tuj-1 were counted and compared relative to the number of astrocytes (GFAP labeled), *n* = 13 chambers per parameter from four isolations. The speed of differentiation was analyzed by counting the number of Sox2 positive cells in the stretched and control cultures after mitogen withdrawal for 5 days, *n* = 3 chambers per parameter from different passages **(C)**. Scale bars in **(A)** and **(B)** = 50 μm, scale bar in **(C)** = 20 μm. *define significant differences (see “Material and Methods” section). ns = non-significant.

We next investigated the influence of cyclic mechanical strain on characteristic functions of NSCs, i.e., proliferation, differentiation, and lineage commitment, respectively. Cyclic stretch for 24 h in the presence of the mitogen FGF significantly reduced the proliferation rate of NSC compared to unstretched controls (17.7% ± 7.8% BrdU positive cells for stretch vs. 29.8% ± 7.6% for control cells, mean ± SD, *p*-value <0.05, Figure 2A).

However, lineage commitment 5 days after mitogen withdrawal to initiate differentiation was not altered by cyclic mechanical stretch that was applied over the entire time (Figure 2B). Furthermore, staining with the stemness marker Sox2 proved that the percentage of still undifferentiated NSCs was not affected by cyclic stretch 5 days after mitogen withdrawal (Figure 2C), suggesting that this mechanical stimulus did neither affect speed nor fate of NSC differentiation.

### Cyclically Stretched NSCs Align Parallel to Cyclic Strain

**Figure 3:**
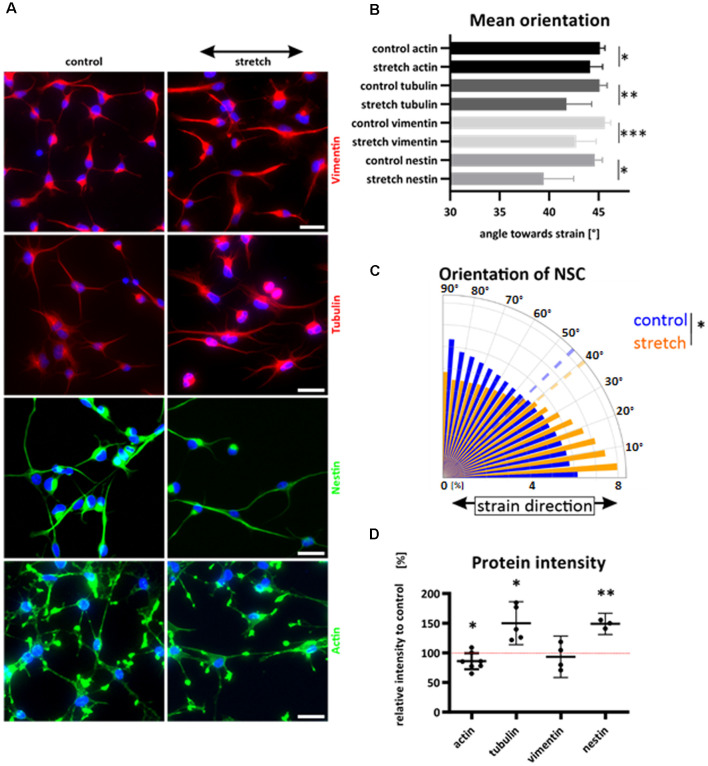
Reorientation of NSCs under strain and cytoskeletal redistribution. NSCs were stretched for 24 h or grown on elastomer chambers as a control. Cells were fixed and cytoskeletal filaments were labeled **(A)**. The black arrow indicates the strain direction. Cyclic stretch induced a reorientation towards smaller angles and direction of strain** (A–C)**. Different cytoskeletal labeling resulted in different mean values of NSC orientation **(B)**, values represent at least *n* = 3 independent experiments with at least *n* = 5 chambers from two different passages. The orientation plot shows the frequency of pixels against nestin and their control relative to their orientation towards strain. The dashed lines show the mean angles **(C)**. Statistical significance was tested by comparing the mean angles of stretch and control (*p*-value = 0.016; 39.5° vs. 44.6°). Relative protein intensity after cyclic stretch **(D)**. Statistics show a one-sample t-test; the hypothetical value of 100% is indicated with a dotted red line. Actin showed a significant reduction under cyclic strain compared to control cells by 14% (*p*-value = 0.0443).The intensity of tubulin increased by 50% (*p*-value = 0.019 and nestin increased when stretched cyclically by 49% (*p*-value = 0.007). Scale bars = 20 μm. *define significant differences (see “Material and Methods” section).

While most mammalian cells align roughly perpendicular to uniaxial cyclic strain to reduce their mechanical stress, we here show for the first time that NSCs aligned parallel to stretch direction (Figures 3A–C). The cytoskeletal filaments of NSCs were differentially spatially distributed within the cell, resulting in a distinct reorientation pattern towards strain (Figure 3B). Here the most significant parallel alignment, with the smallest angle, was observed when cells were stretched and stained against nestin with a mean value of 39.5° for stretched cells vs. 44.6° (*p*-value = 0.016) for unstretched control cells. Nestin was spanning throughout the NSC cell shape as a thin filamentous system, while vimentin and tubulin were more distributed throughout the cell body. However, actin was more localized in peripheral parts and present in cell protrusions, with only a modest realignment (stretch 44.1° vs. 45.13° for controls, *p*-value = 0.041).

Interestingly, we did not observe any stress fiber formation, and actin cytoskeletal distribution was similar in the stretched and control group. In total, cytoskeletal labeling showed a reorientation parallel to strain while exposed to cyclic stretch for 24 h (Figure 3C).

As a semiquantitative approach, we investigated the intensity of cytoskeletal staining on immunofluorescence samples that were stained in parallel and equally illuminated. This way, we identified an increase in image intensity in tubulin and nestin staining in stretched NSC, which indicates a stretch-induced cytoskeletal reinforcement in both cytoskeletal systems (Figure 3D).

### Cyclic Strain During Neural Stem Cell Differentiation Redirects Astrocytes in Stretch Direction While Mechanoresponse Is Lacking in Neuronal Phenotypes

**Figure 4:**
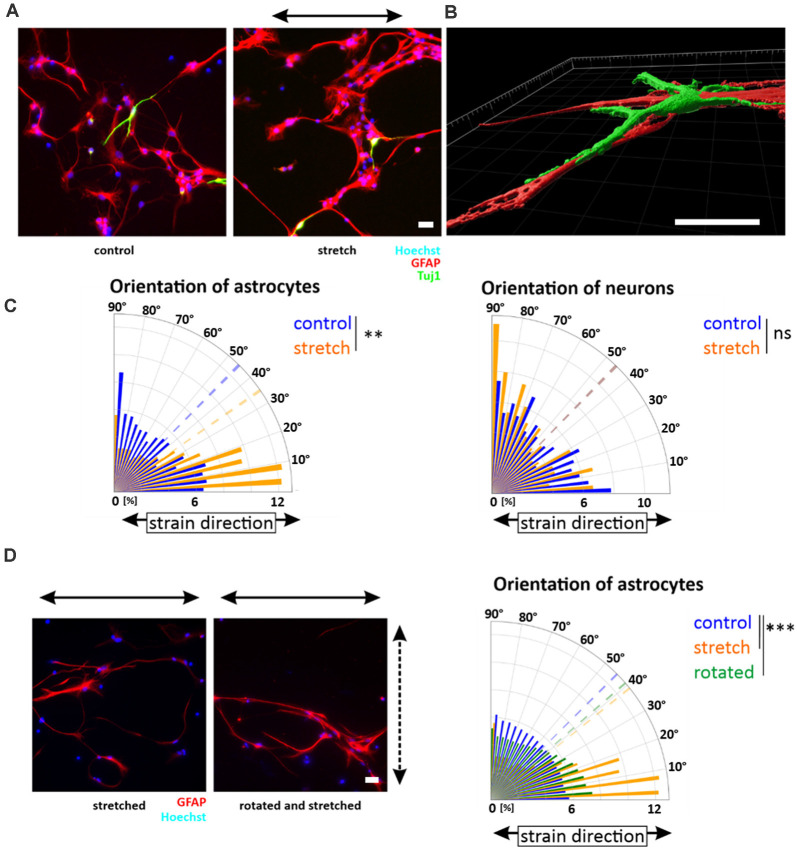
Orientation of NSC-derived young neurons and -astrocytes. Upon mitogen withdrawal, differentiating NSCs were stretched for 5 days or grown on elastomer chambers as a control. After 5 days, cells were fixed and stained against GFAP (astrocytes) and Tuj-1 (neurons) **(A)**. Images were taken with an LSM 880 and processed with the Imaris software **(B)**. Orientation plots are representative plots obtained from pixels stained with GFAP or Tuj-1 and their control **(C)** (mean orientation of stretched young astrocytes vs. control *p*-value = 0.005, *n* = 8 independent experiments from different passages indicated as the dashed line; mean orientations of young neurons in the control and stretch are overlapping). **(D)** After 3 days of stretch, differentiating young astrocyte cultures were removed from their chamber holders and rotated by 90 degrees. The dashed arrow indicates the stretch direction of the first 3 days; the black arrow indicates the stretch direction of the last 2 days. The orientation plot shows the shift of orientation towards smaller angles with significant mean orientations indicated as dotted lines (*p*-value control vs. rotated = 0.0003; *p*-value control vs. stretch = 0.0002, *n* ≥ 8 independent experiments from different passages). Scale bars = 20 μm. *define significant differences (see “Material and Methods” section). ns = non-significant.

Since NSCs reoriented in stretch direction, we next asked how NSCs would respond to strain during differentiation induced by mitogen withdrawal. Similar to the observation in undifferentiated NSC, NSC-derived young astrocytes aligned parallel to cyclic strain (Figure 4A). Quantitative analyses of astrocytes stretched during differentiation revealed a clear shift in GFAP alignment toward strain (mean angle 34.8 ± 7°) compared to a random distribution for unstretched control cells (45.4 ± 5°, *p*-value <0.01, Figure 4C). In contrast, NSC-derived young neurons did not show any reorientation but a steady random filament orientation. Analysis by confocal microscopy and 3D reconstruction revealed that young neurons in these mixed differentiation cultures grew on top of astrocytes (Figure 4B).

To investigate if the reorientation behavior was an active, persisting process of differentiating astrocytes, the direction of uniaxial strain was rotated by 90° after 3 days of stretch, a timepoint when directional outgrowth was already visible. Rotation artificially forced astrocytes into the direction away from the stretch. However, ongoing cyclic stretch for additional 2 days restored the astrocytes’ parallel orientation (Figure 4D), highlighting that alignment in strain direction characterized a stable and active mechanoresponse of young astrocytes to cyclic strain.

### RhoA Activation in Orientation Towards Cyclic Strain in NSCs

**Figure 5:**
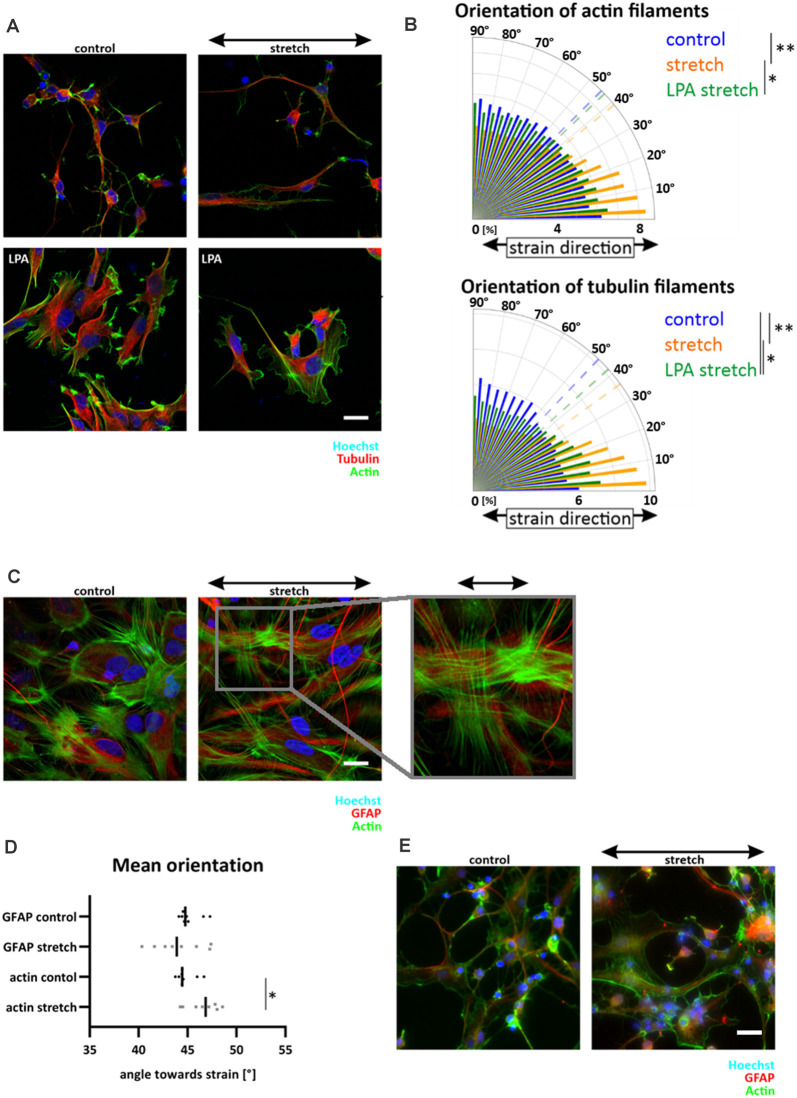
RhoA activation in NSC reduced parallel orientation towards strain. NSCs were stretched for 24 h with an amplitude of 15% and 300 mHz, 40 min before fixation cells were incubated with 30 μM LPA **(A)**. The orientation plot shows the fiber distribution of control cells, stretched cells and stretched with activated RhoA **(B)**, actin filament distribution: *p*-value LPA stretch vs. stretch = 0.0173; *p*-value stretch vs. control = 0.0022; tubulin filament distribution: *p*-value stretch vs. control = 0.00173; *p*-value LPA stretch vs. control = 0.0173; *p*-value stretch vs. control = 0.0022, *n* ≥ 5 independent experiments from three different passages. Postnatal astrocytes on elastomer chambers showed stress fibers in control cells or when stretched for 24 h with the same stretch parameter **(C)**. GFAP cytoskeleton showed a random distribution **(D)** while actin staining revealed significant stress fiber formation perpendicular to strain (*p*-value = 0.0379, *n* = 8 independent experiments from three different passages). Stress fibers were not present in differentiating astrocytes **(E)**. Here NSCs were stretched during their mitogen withdrawal and fixed after 5 days of differentiation. Scale bars = 20 μm. *define significant differences (see “Material and Methods” section).

A parallel alignment of cells to strain has previously been described as dependent on the activation of the GTPase RhoA, and associated with a lack of stress fibers in endothelial and osteosarcoma cells (Lee et al., 2010; Tondon and Kaunas, 2014). As we observed a lack of central stress fibers in NSCs (cf. Figure 3A), we hypothesized that a lack of contractility within NSCs might be responsible for aligning these cells in the direction of maximal mechanical loads. To test this hypothesis, we increased NSCs contractility by activation of RhoA with lysophosphatidic acid (LPA) and observed a clear formation of central stress fibers in NSCs independent of strain (Figure 5A). Furthermore, upon stretch application, stress fiber induction significantly reduced NSCs reorientation behavior in direction of maximal mechanical load, therefore decreasing the previously found mechanoresponse of NSCs (Figure 5B). These results were corroborated by missing stress fibers in differentiating young astrocytes, which instead display a random distribution of a thin actin meshwork (Figure 5E).

In contrast, stress fibers were present in postnatal astrocytes. Here, a slight reorientation in perpendicular direction upon stretch could be detected (Figures 5C,D, mean angle 46.6 ± 1.6) compared to unstretched cells (44.8 ± 1, *p*-value = 0.038). Notably, the GFAP cytoskeleton of astrocytes was unaffected by cyclic stretch. It did not show any preferred direction after 24 h of stretch, leading to randomly distributed long cell shapes of postnatal astrocytes that reacted to cyclic deformations only with their actin cytoskeleton (Figure 5D).

### Mechanoprotective Role of Postnatal Astrocytes

**Figure 6:**
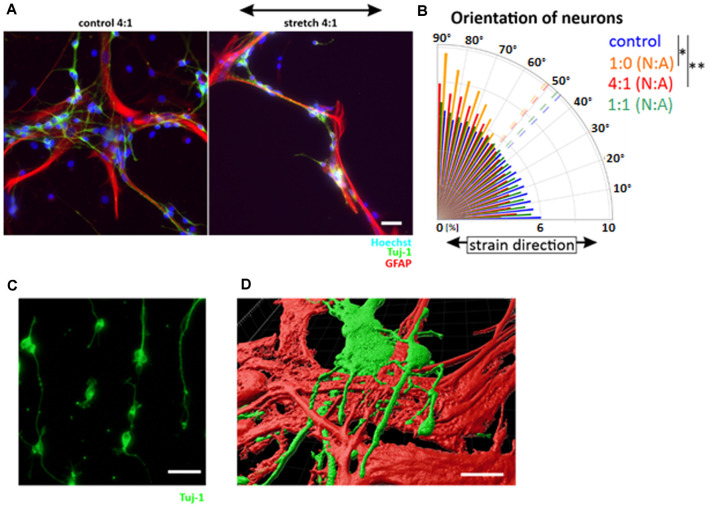
Mechanoprotective effect of postnatal astrocytes. Cocultures of neuronal cells and astrocytes were produced by cultivating cortical neurons and postnatal astrocytes in different ratios and stretched for 24 h **(A)**. The orientation plot shows different degrees of reorientation depending on the number of astrocytes in the coculture **(B)**. Statistics was performed comparing the mean orientation: 4:1 neurons (N): astrocytes **(A)** *p*-value = 0.002, 1:0 N:A *p*-value = 0.022, *n* ≥ 3 independent experiments, from four different passages (mean values indicated as dashed lines). Cortical neurons stretched with the same parameters show a perpendicular orientation to stretch **(C)**. 3D view of neurons on top of astrocytes **(D)**. Scale bars = 20 μm. *define significant differences (see “Material and Methods” section).

To further elucidate the differences observed between astrocytes and neurons as well as across differentiation stages, we additionally assessed the response to cyclic strain in an astrocyte/neuron coculture with varying, yet predefined, cell ratios (Figures 6A,B). While isolated neurons aligned in perpendicular direction to strain (Figures 6B,C), realignment of neurons in coculture was dependent on the proportion of astrocytes (Figure 6B). With increasing numbers of astrocytes, alignment of neurons continuously decreased leading to a random orientation of neuronal cells when astrocytes were plated with the same cell number (1:1). However, when fewer astrocytes were in the coculture (4:1), neuronal cells kept their mechanoresponse with an alignment in perpendicular direction relative to strain. Confocal microcopy and subsequent 3D reconstruction revealed that cortical neurons grew on postnatal astrocytes in mixed cultures (Figure 6D). These data do not only confirm cooperative cell growth as shown before for differentiating astrocytes and neurons (Figure 4B), but also argue for a mechanoprotective function of astrocytes for differentiated neurons.

We further analyzed this hypothesis in stretch experiments on neurons and cocultures of neurons and astrocytes in the presence of LPA. These data confirm the reorientation of pure neurons in perpendicular direction relative to stretch. Interestingly, presence of astrocytes in a 1–1 ratio preserves their mechanoprotective effect on neurons as shown above even with activated RhoA. This effect remained stable independent if RhoA was activated during the whole stretching time of 24 h or just for the last 40 min of stretching (Supplementary Figure 1).

## Discussion

Mechanical cues such as tissue strain in particular, have an important, yet often neglected impact on brain cells (Tavazoie et al., 2008; for review see: Gangatharan et al., 2018). In the mice’s stem cell niche, blood vessel pulsation might lead to tissue displacement of up to 30% (Tavazoie et al., 2008; Drew et al., 2011). As most studies on the physical microenvironment focus on stiffness alterations and topographical cues, the effects of this pulsatile strain remain to be delineated. We here investigated the effect of mechanical strain on cells within the NSC niche. We observed that NSCs were affected by mechanical strain at the morphological level and concerning their proliferative activity. Interestingly, NSC and their young astrocytic progeny actively oriented in strain direction, sheltering the surrounding (young) neurons from mechanical impact. These novel findings highlight that both NSCs and their young astrocytic progeny respond fundamentally differently to cyclic strain than other mammalian cells (Faust et al., 2011), including neurons (Abraham et al., 2019), which preferentially orient perpendicular to the strain direction, as to actively escape the mechanical stress.

Mechanical forces are present during brain development. Thus, we propose that a distinct response to mechanical cues based on their differentiation and developmental status is of pivotal importance during developmental processes of the brain. Notably, directional cellular organizations are formed during early development, e.g., radial glia cells projecting their axons from the ventricular zone to the cortical plate, and thereby supplying topographical support and guidance for migrating neural precursor cells. In adulthood, such radial glia cells develop into stellate astrocytes and neurons (Leavitt et al., 1999).

Despite biochemical signals, mechanical cues such as pressure, stiffness, stretch, or topography can tune cell behavior during embryogenesis (Keller et al., 2003). Stiffness and topography are crucial regulators of stem cell characteristics, e.g., lineage commitment and proliferation (Baek et al., 2018; Blaschke et al., 2019). Here, we report the intriguing finding that NSCs apparently do not only sense pulsatile strain—indicated by their active re-orientation—but also align towards this strain, unlike other known mammalian cells. It is yet unknown why NSCs chose to potentiate this mechanical stress by orienting in stretch direction, but we speculate that it might be one of the factors to keep them in a quiescent state within the NSC niche. In a previous study, we had reported that soft tissue culture substrates mimicking the brain’s elasticity promote quiescence in NSCs (Blaschke et al., 2019). Corroborating those results, we here report a significantly decreased NSC proliferation rate, without signs of cell impairment, suggesting induction of cell quiescence in response to cyclic strain. Quiescence is a vital characteristic of NSCs as it provides a mechanism to exist in an undifferentiated state, preserving the progenitor pool. Still, quiescent cells remain responsive to external stimuli, e.g., during damage or degeneration (Wang et al., 2011). This way, we suggest an alternative or additive mode of preservation of NSC stemness within the niche, besides a reported direct cell-cell interaction with endothelial cells (Ottone et al., 2014). Supporting our hypothesis, a study by Paul and colleagues also observed a reduced proliferation rate when they cyclically stretched human adipose-derived stem cells (Paul et al., 2018). Their study’s reduced proliferation rate was related to G_2_/M phase cell cycle arrest and linked to a decreased extracellular signal-regulated kinase (ERK) 1/2 and histone H3 phosphorylation.

Previous reports suggested lineage commitment of differentiating NSCs to be affected by mechanical stimuli, i.e., elasticity (Blaschke et al., 2019) or static stretch (Arulmoli et al., 2015). On elastomers with different substrate stiffness, we previously reported an altered NSCs lineage commitment by promoting neurogenesis on brain-like elasticities (Blaschke et al., 2019). Likewise, Arulmoli et al. reported a reduction in oligodendrogenesis by application of 10% static stretch on mice NSCs (Arulmoli et al., 2015). Interestingly, in our study, mechanical strain did not influence lineage choice between astrocytes and neuronal cells when exposed to cyclic stretch over 5 days. Such an unaffected behavior would fit well with a continuously present mechanical signal to allow regulation of differentiation by higher-level mechanisms *in vivo*, as e.g., transcription factor regulation (Pataskar et al., 2016), cell-cell communication (Tsai and Mckay, 2000), and soluble differentiation factors (Dumont et al., 2017).

Alterations on different elasticities are associated with a different cytoskeletal reorganization, altered F-actin structures, and altered focal adhesion formation (Baek et al., 2018). In this study, we did not observe a different cytoskeletal actin reorganization induced by stretching that might explain the observed independence of NSCs lineage commitment from a cyclic strain. Still an independence of NSC lineage commitment from mechanical signals was not expected as NSCs indeed respond to mechanical disturbances such as stiffness (Blaschke et al., 2019).

Notably, we observed a cytoskeletal reinforcement. However, the overall cytoskeletal architecture remained unaffected between the stretched and control cells. Thus, we assumed that substrate stiffness as a mechanical cue may influence the NSC cytoskeletal architecture more than mechanical strain. Moreover, NSCs grown on 50 kPa elastomers were subjected to a higher mechanical stiffness as found in their natural physical microenvironment (Engler et al., 2006), thus cells may be already affected by an unnatural, stiffer microenvironment.

Generally, when mammalian cells are exposed to cyclic strain, they align in perpendicular directions relative to the strain (Faust et al., 2011). Among cytoskeletal filaments, the actin cytoskeleton is the first to orient, followed by microtubule and intermediate filaments and the overall cell shape (Zielinski et al., 2018; Springer et al., 2019). In this study, actin filament’s cytoskeletal distribution of actin filaments in stretched NSCs did not differ from cells grown on elastomer chambers without stretch. Moreover, with NSCs and differentiating astrocytes, we observed contrary reorientation behavior compared to other cell types, with cyclically stretched NSCs aligning in the direction of maximal mechanical loading, parallel to stretch. Furthermore, we witnessed a likewise behavior in young astrocytes during their differentiation. Most interestingly, the alignment of astrocytes dynamically changed when the stretch direction was rotated orthogonally. A lack of stress fiber formation was observed in both cell types that aligned parallel to stretch- in NSCs and young astrocytes.

An orientation parallel to stretch was described for quasi-static or static strain (Eastwood et al., 1998; Collinsworth et al., 2000; De and Safran, 2008; Morioka et al., 2011; Xu et al., 2018). Interestingly, also RhoA inhibition induced stress fiber formation in parallel orientation after uniaxial cyclic stretching in another study by Kaunas and colleagues (Kaunas et al., 2005). Furthermore, another study confirmed such data (Lee et al., 2010), mediated by its interference with central stress fiber formation and parallel orientation of remaining stress fibers that were located in the cell’s periphery. In line with these reports, NSCs here did not reveal any stress fibers, while actin bundles were only observed in the cell periphery and mostly within cell protrusions. A reduced formation of stress fibers was also observed in Tondon and colleagues’ study, which observed a parallel alignment of human osteosarcoma cells when stretched on very soft structures (Tondon and Kaunas, 2014). Thus, the mechanism for parallel alignment may be similar across cell types. The authors argue that the driving force may be an optimal cellular tension that drives the cell to orient parallel to strain. However, the exact mechanism of parallel alignment during a cyclic stretch remain unclear. One critical GTPase that links the absence of stress fibers and impeded reorientation is RhoA, which was described as an essential player in the reorientation process to cyclic strain (Goldyn et al., 2009). Furthermore it is also known as important regulator for dendritic arborization, spine morphogenesis, growth cone development, and axon guidance upon activation in neurons (Stankiewicz and Linseman, 2014). In agreement with this, when LPA induced RhoA activity, we found a de novo stress fiber formation and a simultaneous loss of NSCs reorientation behavior in stretch direction. Based on critical strain thresholds in the range of 3–4% in the presence of actin bundles (Kirchenbuchler et al., 2010; Faust et al., 2011), we suggest that low RhoA activity in NSCs might be responsible for the absence of stress fibers and cell alignment—however, further research is warranted to elucidate this matter.

Upon mitogen withdrawal, NSCs differentiate into a coculture of young neurons and glia. Interestingly, we observed differences in the mechanoresponses to a cyclic stretch of those cell types. While astrocytes exhibited a distinct reorientation parallel to the strain, stretched neuronal cells remained randomly distributed. Furthermore, a 3D analysis revealed that neuronal cells grow on top of differentiating astrocytes. Thus, we hypothesize that neuronal cells avoid mechanical stress and might be protected from mechanical strain by underlying astrocytes. We could show that this effect seems to be preserved even when RhoA is activated.

Thus, due the soft nature of astrocytes (Lu et al., 2006; Moeendarbary et al., 2017) and also the increased spatial distance of neurons to the deforming elastomer substrate, the mechanical impact on neuronal cells is likely diminished. In line with this, we showed that the ratio of astrocytes and neurons essentially determined the degree of neuronal alignment. For optimal comparison, we used the same experimental setup as in our previous study, where we analyzed how cortical neurons respond to mechanical strain in detail (Abraham et al., 2019).

Conjointly, these findings might highlight the astrocytes’ vital role in scavenging the mechanical impact on neuronal phenotypes and their proposed mechanoprotective function. Such a mechanoprotection fits well with the astrocytes’ role of supporting, guiding, and enhancing neuronal growth (East et al., 2010). Likewise, a close interaction of neuronal cells and astrocytes influences astrocytic metabolism and gene transcription (Hasel et al., 2017). Furthermore, astrocytes closely interact with blood vessels and thereby even regulate the blood flow and vascular diameter (Sofroniew and Vinters, 2010), thus possibly also buffering the mechanical load of the brain vasculature.

Altogether, our findings emphasize the importance of universal mechanical forces on fundamental brain cell properties both in developmental and adult stages and give insight into the complex yet unknown mechanisms of NSCs behavior in the neurogenic niche.

## Data Availability Statement

The raw data supporting the conclusions of this article will be made available by the authors, without undue reservation.

## Author Contributions

MR, J-AA, and BH designed research. J-AA, ST, and SB performed research. GD supported research by providing software for analysis. MS, BH, SV, MS, GF, and RM discussed the results. MR, J-AA, and BH wrote the article. Manuscript corrections were done by all authors. All authors contributed to the article and approved the submitted version.

## Conflict of Interest

The authors declare that the research was conducted in the absence of any commercial or financial relationships that could be construed as a potential conflict of interest.

## Publisher’s Note

All claims expressed in this article are solely those of the authors and do not necessarily represent those of their affiliated organizations, or those of the publisher, the editors and the reviewers. Any product that may be evaluated in this article, or claim that may be made by its manufacturer, is not guaranteed or endorsed by the publisher.

## Supplementary Materials

SUPPLEMENTARY FIGURE 1 Neuronal reorientation in pure and coculture of astrocytes and neuronal cells with RhoA activation. Neurons alone and mixed cultures of neurons and astrocytes (1:1 ratio) were grown and stretched for 24 h in the presence of LPA (30 μM). Subsequently, cells were stained for actin after fixation and analyzed for main cell orientation. For optimal comparison data from Figure 6 on same cultures in the absence of LPA are indicated as well. For statistical analysis mean distribution angles were compared. **p*-value below 0.05 **(A)**. Same experiments were performed with induction of RhoA for just the last 40 min of cyclic stretching and subsequently compared with the 24 h data shown in A. For those data sets color coding was kept the same **(B)**.

## References

[B1] Abraham J.-A. Linnartz C. Dreissen G. Springer R. Blaschke S. Rueger M. A.. (2019). Directing neuronal outgrowth and network formation of rat cortical neurons by cyclic substrate stretch. Langmuir 35, 7423–7431. 10.1021/acs.langmuir.8b02003 30110535

[B2] Arulmoli J. Pathak M. M. Mcdonnell L. P. Nourse J. L. Tombola F. Earthman J. C.. (2015). Static stretch affects neural stem cell differentiation in an extracellular matrix-dependent manner. Sci. Rep. 5:8499. 10.1038/srep08499 25686615 PMC4330529

[B3] Baek J. Cho S. Y. Kang H. Ahn H. Jung W. B. Cho Y.. (2018). Distinct mechanosensing of human neural stem cells on extremely limited anisotropic cellular contact. ACS Appl. Mater. Interfaces 10, 33891–33900. 10.1021/acsami.8b10171 30207452

[B4] Blaschke S. Vay S. U. Pallast N. Rabenstein M. Abraham J.-A. Linnartz C.. (2019). Substrate elasticity induces quiescence and promotes neurogenesis of primary neural stem cells—A biophysical *in vitro* model of the physiological cerebral milieu. J. Tissue Eng. Regen. Med. 13, 960–972. 10.1002/term.2838 30815982

[B5] Collinsworth A. M. Torgan C. E. Nagda S. N. Rajalingam R. J. Kraus W. E. Truskey G. A. (2000). Orientation and length of mammalian skeletal myocytes in response to a unidirectional stretch. Cell Tissue Res. 302, 243–251. 10.1007/s004410000224 11131135

[B6] De R. Safran S. A. (2008). Dynamical theory of active cellular response to external stress. Phys. Rev. E Stat. Nonlin. Soft Matter Phys. 78:031923. 10.1103/PhysRevE.78.031923 18851081

[B7] Drew P. J. Shih A. Y. Kleinfeld D. (2011). Fluctuating and sensory-induced vasodynamics in rodent cortex extend arteriole capacity. Proc. Natl. Acad. Sci. U S A 108, 8473–8478. 10.1073/pnas.1100428108 21536897 PMC3100929

[B8] Dumont C. M. Piselli J. M. Kazi N. Bowman E. Li G. Linhardt R. J.. (2017). Factors released from endothelial cells exposed to flow impact adhesion, proliferation and fate choice in the adult neural stem cell lineage. Stem Cells Dev. 26, 1199–1213. 10.1089/scd.2016.0350 28557666 PMC5564022

[B9] East E. De Oliveira D. B. Golding J. P. Phillips J. B. (2010). Alignment of astrocytes increases neuronal growth in three-dimensional collagen gels and is maintained following plastic compression to form a spinal cord repair conduit. Tissue Eng. Part A 16, 3173–3184. 10.1089/ten.tea.2010.0017 20649441 PMC2958448

[B10] Eastwood M. Mudera V. Mcgrouther D. Brown R. (1998). Effect of precise mechanical loading on fibroblast populated collagen lattices: morphological changes. Cell Motil. Cytoskeleton 40, 13–21. 10.1002/(SICI)1097-0169(1998)40:1<13::AID-CM2>3.0.CO;2-G 9605968

[B11] Engler A. J. Sen S. Sweeney H. L. Discher D. E. (2006). Matrix elasticity directs stem cell lineage specification. Cell 126, 677–689. 10.1016/j.cell.2006.06.044 16923388

[B13] Faust U. Hampe N. Rubner W. Kirchgessner N. Safran S. Hoffmann B.. (2011). Cyclic stress at mHz frequencies aligns fibroblasts in direction of zero strain. PloS One 6:e28963. 10.1371/journal.pone.0028963 22194961 PMC3241701

[B14] Fletcher D. A. Mullins R. D. (2010). Cell mechanics and the cytoskeleton. Nature 463, 485–492. 10.1038/nature08908 20110992 PMC2851742

[B15] Gangatharan G. Schneider-Maunoury S. Breau M. A. (2018). Role of mechanical cues in shaping neuronal morphology and connectivity. Biol. Cell 110, 125–136. 10.1111/boc.201800003 29698566

[B16] Goldberg J. S. Hirschi K. K. (2009). Diverse roles of the vasculature within the neural stem cell niche. Regen. Med. 4, 879–897. 10.2217/rme.09.61 19903006 PMC2836203

[B17] Goldyn A. M. Rioja B. A. Spatz J. P. Ballestrem C. Kemkemer R. (2009). Force-induced cell polarisation is linked to RhoA-driven microtubule-independent focal-adhesion sliding. J. Cell Sci. 122, 3644–3651. 10.1242/jcs.054866 19812308 PMC2758800

[B18] Hasel P. Dando O. Jiwaji Z. Baxter P. Todd A. C. Heron S.. (2017). Neurons and neuronal activity control gene expression in astrocytes to regulate their development and metabolism. Nat. Commun. 8:15132. 10.1038/ncomms15132 28462931 PMC5418577

[B19] Karakatsani A. Shah B. Ruiz De Almodovar C. (2019). Blood vessels as regulators of neural stem cell properties. Front. Mol. Neurosci. 12:85. 10.3389/fnmol.2019.00085 31031591 PMC6473036

[B20] Kaunas R. Nguyen P. Usami S. Chien S. (2005). Cooperative effects of Rho and mechanical stretch on stress fiber organization. Proc. Natl. Acad. Sci. U S A 102, 15895–15900. 10.1073/pnas.0506041102 16247009 PMC1276069

[B21] Keller R. Davidson L. A. Shook D. R. (2003). How we are shaped: the biomechanics of gastrulation. Differentiation 71, 171–205. 10.1046/j.1432-0436.2003.710301.x 12694202

[B22] Kirchenbuchler D. Born S. Kirchgessner N. Houben S. Hoffmann B. Merkel R. (2010). Substrate, focal adhesions and actin filaments: a mechanical unit with a weak spot for mechanosensitive proteins. J. Phys. Condens. Matter 22:194109. 10.1088/0953-8984/22/19/194109 21386436

[B23] Leavitt B. R. Hernit-Grant C. S. Macklis J. D. (1999). Mature astrocytes transform into transitional radial glia within adult mouse neocortex that supports directed migration of transplanted immature neurons. Exp. Neurol. 157, 43–57. 10.1006/exnr.1999.6982 10222107

[B24] Lee C. F. Haase C. Deguchi S. Kaunas R. (2010). Cyclic stretch-induced stress fiber dynamics - dependence on strain rate, Rho-kinase and MLCK. Biochem. Biophys. Res. Commun. 401, 344–349. 10.1016/j.bbrc.2010.09.046 20849825

[B25] Lu Y.-B. Franze K. Seifert G. Steinhäuser C. Kirchhoff F. Wolburg H.. (2006). Viscoelastic properties of individual glial cells and neurons in the CNS. Proc. Natl. Acad. Sci. U S A 103, 17759–17764. 10.1073/pnas.0606150103 17093050 PMC1693820

[B26] Miller F. D. Gauthier-Fisher A. (2009). Home at last: neural stem cell niches defined. Cell Stem Cell 4, 507–510. 10.1016/j.stem.2009.05.008 19497279

[B27] Moeendarbary E. Weber I. P. Sheridan G. K. Koser D. E. Soleman S. Haenzi B.. (2017). The soft mechanical signature of glial scars in the central nervous system. Nat. Commun. 8:14787. 10.1038/ncomms14787 28317912 PMC5364386

[B28] Morioka M. Parameswaran H. Naruse K. Kondo M. Sokabe M. Hasegawa Y.. (2011). Microtubule dynamics regulate cyclic stretch-induced cell alignment in human airway smooth muscle cells. PLoS One 6:e26384. 10.1371/journal.pone.0026384 22022610 PMC3195692

[B29] Noethel B. Ramms L. Dreissen G. Hoffmann M. Springer R. Rübsam M.. (2018). Transition of responsive mechanosensitive elements from focal adhesions to adherens junctions on epithelial differentiation. Mol. Biol. Cell 29, 2317–2325. 10.1091/mbc.E17-06-0387 30044710 PMC6249805

[B30] Otsuki L. Brand A. H. (2017). The vasculature as a neural stem cell niche. Neurobiol. Dis. 107, 4–14. 10.1016/j.nbd.2017.01.010 28132930

[B31] Ottone C. Krusche B. Whitby A. Clements M. Quadrato G. Pitulescu M. E.. (2014). Direct cell-cell contact with the vascular niche maintains quiescent neural stem cells. Nat. Cell Biol. 16, 1045–1056. 10.1038/ncb3045 25283993 PMC4298702

[B32] Pataskar A. Jung J. Smialowski P. Noack F. Calegari F. Straub T.. (2016). NeuroD1 reprograms chromatin and transcription factor landscapes to induce the neuronal program. EMBO J. 35, 24–45. 10.15252/embj.201591206 26516211 PMC4718003

[B33] Paul N. E. Denecke B. Kim B. S. Dreser A. Bernhagen J. Pallua N. (2018). The effect of mechanical stress on the proliferation, adipogenic differentiation and gene expression of human adipose-derived stem cells. J. Tissue Eng. Regen. Med. 12, 276–284. 10.1002/term.2411 28095649

[B34] Rueger M. A. Backes H. Walberer M. Neumaier B. Ullrich R. Simard M.-L.. (2010). Noninvasive imaging of endogenous neural stem cell mobilization *in vivo* using positron emission tomography. J. Neurosci. 30, 6454–6460. 10.1523/JNEUROSCI.6092-09.2010 20445071 PMC6632716

[B35] Sofroniew M. V. Vinters H. V. (2010). Astrocytes: biology and pathology. Acta Neuropathol. 119, 7–35. 10.1007/s00401-009-0619-8 20012068 PMC2799634

[B36] Springer R. Zielinski A. Pleschka C. Hoffmann B. Merkel R. (2019). Unbiased pattern analysis reveals highly diverse responses of cytoskeletal systems to cyclic straining. PLoS One 14:e0210570. 10.1371/journal.pone.0210570 30865622 PMC6415792

[B37] Srivastava N. Venugopalan V. Divya M. S. Rasheed V. A. James J. Narayan K. S. (2013). Neuronal differentiation of embryonic stem cell derived neuronal progenitors can be regulated by stretchable conducting polymers. Tissue Eng. Part A 19, 1984–1993. 10.1089/ten.TEA.2012.0626 23544950 PMC3725875

[B38] Stankiewicz T. R. Linseman D. A. (2014). Rho family GTPases: key players in neuronal development, neuronal survival and neurodegeneration. Front. Cell. Neurosci. 8:314. 10.3389/fncel.2014.00314 25339865 PMC4187614

[B39] Tavazoie M. Van Der Veken L. Silva-Vargas V. Louissaint M. Colonna L. Zaidi B.. (2008). A specialized vascular niche for adult neural stem cells. Cell Stem Cell 3, 279–288. 10.1016/j.stem.2008.07.025 18786415 PMC6864413

[B40] Tondon A. Kaunas R. (2014). The direction of stretch-induced cell and stress fiber orientation depends on collagen matrix stress. PloS One 9:e89592. 10.1371/journal.pone.0089592 24586898 PMC3933569

[B41] Tsai R. Y. Mckay R. D. (2000). Cell contact regulates fate choice by cortical stem cells. J. Neurosci. 20, 3725–3735. 10.1523/JNEUROSCI.20-10-03725.2000 10804214 PMC6772699

[B42] Ulbricht A. Eppler F. J. Tapia V. E. Van Der Ven P. F. Hampe N. Hersch N.. (2013). Cellular mechanotransduction relies on tension-induced and chaperone-assisted autophagy. Curr. Biol. 23, 430–435. 10.1016/j.cub.2013.01.064 23434281

[B43] Wang Y.-Z. Plane J. M. Jiang P. Zhou C. J. Deng W. (2011). Concise review: quiescent and active states of endogenous adult neural stem cells: identification and characterization. Stem Cells 29, 907–912. 10.1002/stem.644 21557389 PMC3306660

[B44] Xu G.-K. Feng X.-Q. Gao H. (2018). Orientations of cells on compliant substrates under biaxial stretches: a theoretical study. Biophys. J. 114, 701–710. 10.1016/j.bpj.2017.12.002 29414715 PMC5985023

[B45] Yokota Y. Kim W. Y. Chen Y. Wang X. Stanco A. Komuro Y.. (2009). The adenomatous polyposis coli protein is an essential regulator of radial glial polarity and construction of the cerebral cortex. Neuron 61, 42–56. 10.1016/j.neuron.2008.10.053 19146812 PMC2804250

[B46] Zielinski A. Linnartz C. Pleschka C. Dreissen G. Springer R. Merkel R.. (2018). Reorientation dynamics and structural interdependencies of actin, microtubules and intermediate filaments upon cyclic stretch application. Cytoskeleton (Hoboken) 75, 385–394. 10.1002/cm.21470 30176121

