# Supplementary figures and images for: NSCs Under Strain—Unraveling the Mechanoprotective Role of Differentiating Astrocytes in a Cyclically Stretched Coculture With Differentiating Neurons
Source: Front Cell Neurosci. 2021 Sep 24;15:706585. doi: 10.3389/fncel.2021.706585 (PMC8497758; doi:10.3389/fncel.2021.706585)

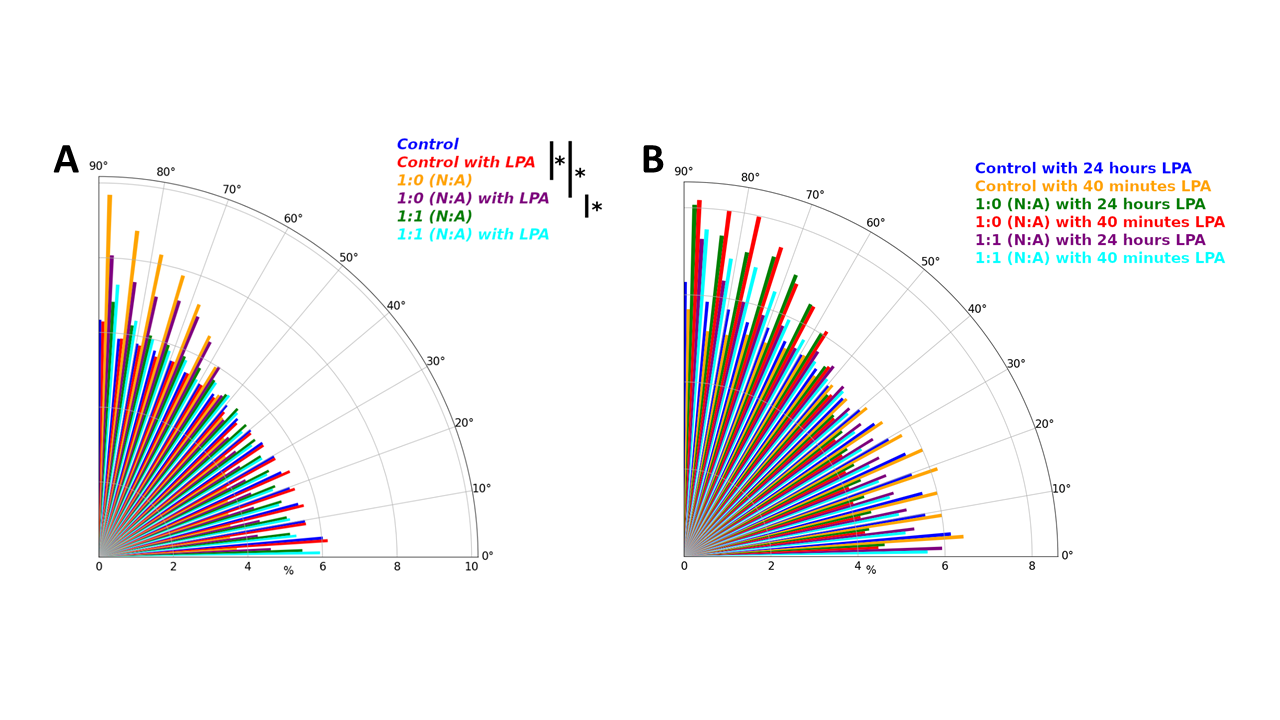

Supplement: SUPPLEMENTARY FIGURE 1 — Neuronal reorientation in pure and coculture of astrocytes and neuronal cells with RhoA activation. Neurons alone and mixed cultures of neurons and astrocytes (1:1 ratio) were grown and stretched for 24 h in the presence of LPA (30 μM). Subsequently, cells were stained for actin after fixation and analyzed for main cell orientation. For optimal comparison data from Figure 6 on same cultures in the absence of LPA are indicated as well. For statistical analysis mean distribution angles were compared. *p-value below 0.05 (A). Same experiments were performed with induction of RhoA for just the last 40 min of cyclic stretching and subsequently compared with the 24 h data shown in A. For those data sets color coding was kept the same (B). [file Image_1.TIF]
